# Prevalence of canine heartworm infection in Queensland, Australia: comparison of diagnostic methods and investigation of factors associated with reduction in antigen detection

**DOI:** 10.1186/s13071-022-05633-9

**Published:** 2023-02-10

**Authors:** Constantin Constantinoiu, Catriona Croton, Mandy B. A. Paterson, Lyn Knott, Joerg Henning, John Mallyon, Glen T. Coleman

**Affiliations:** 1 James Cook University, Townsville, QLD Australia; 2 University of Southern Queensland, Toowoomba, QLD Australia; 3 Royal Society for the Prevention of Cruelty to Animals, Brisbane, QLD Australia; 4 The University of Queensland, Gatton, QLD Australia

**Keywords:** Dirofilaria immitis, Dogs, Prevalence, Diagnostic test, Antigen detection, Dissociation of immune complexes

## Abstract

**Background:**

The prevalence of *Dirofilaria immitis* infection in dogs is increasing globally and spreading into new areas. Prevalence of dirofilariosis in the state of Queensland, Australia, was as high as 90% before the introduction of macrocyclic lactones. Limited research on prevalence of *D. immitis* infection in dogs in Queensland has been reported in the last 30 years. Antigen testing is the most common method for detection of dirofilariosis but its accuracy is reduced by antigen getting trapped (blocked antigen) in immune complexes (ICs). The objectives of this research were to determine the prevalence of *D. immitis* infection in dogs from two geographical areas (Brisbane and Townsville) in Queensland, to determine the extent to which blocked antigen affects the validity of antigen testing, and to explore whether this was associated with microfilaraemia, location, age or sex.

**Methods:**

Blood samples from Brisbane (sub-tropical climate) and Townsville (tropical climate) shelter dogs were evaluated for the presence of *D. immitis* antigen before (conventional antigen testing, CAT) and after dissociation of ICs by heat treatment (antigen testing after heat treatment, ATHT), using a commercially available test. Microfilariae were detected using modified Knott’s test (MKT). Test proportions were compared with McNemar’s test and the association between antigen test-discordant results (positive for antigen after dissociation of ICs) and microfilaraemia, location, sex and age was modelled using logistic regression.

**Results:**

*Dirofilaria immitis* prevalence in dogs from Townsville (22% by CAT, 32.1% by ATHT and 16.7% by MKT) was significantly higher than in dogs from Brisbane (1.1% by CAT and MKT and 1.7% by ATHT) $(P< 0.001)$. Dissociation of ICs allowed detection of significantly more *D. immitis* infected dogs than either conventional antigen testing or microfilariae detection, or the combined antigen and microfilariae detection $(P< 0.001)$. The odds of dogs being positive for antigen after dissociation of ICs were significantly higher for microfilaraemic, 3–4-year-old female dogs from Townsville.

**Conclusions:**

The high prevalence of infection with *D. immitis* in dogs from Townsville poses a health risk for local susceptible host species, including humans. Dissociation of ICs increases antigen detection and should be considered in dogs suspected of *D. immitis* infection but negative on routine testing.

**Graphical Abstract:**

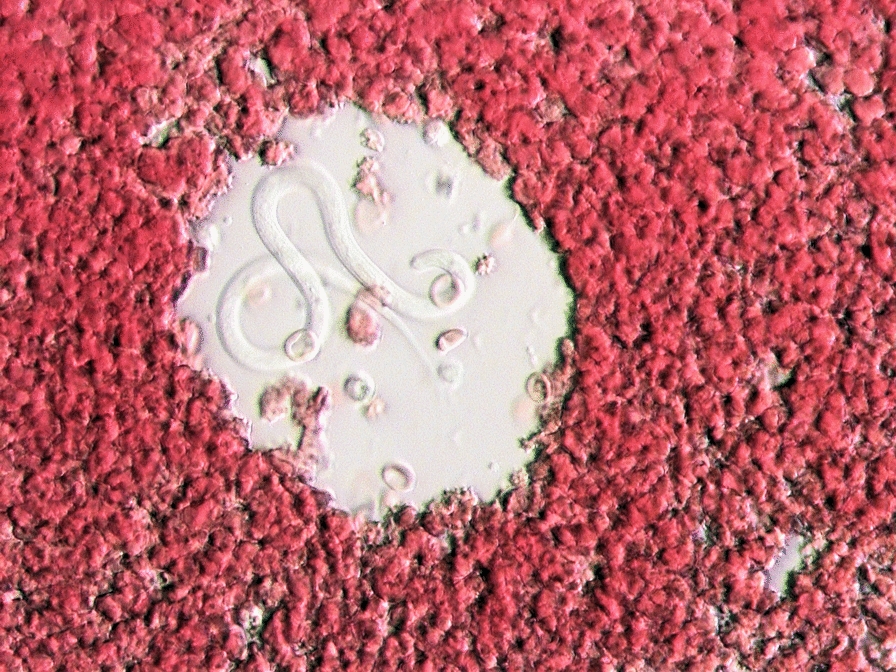

## Background

Canine heartworm infection (HWI) is a life-threatening condition caused by infection with *Dirofilaria immitis*, a mosquito-borne, filarioid nematode. Infection with *D. immitis* is geographically widespread, but prevalence and worm burden in tropical and subtropical areas are usually higher than in temperate areas [1, 2]. The prevalence of infection with *D. immitis* depends on many factors including distribution and density of the mosquito intermediate hosts, precipitation, relative humidity, elevation, age of the host, owner compliance with preventive measures, socio-economic status of dog owners, presence of wildlife reservoirs, etc. [3, 4]. Worldwide, the prevalence of HWI in dogs appears to have declined over the last few years [5]. However, currently, two opposing tendencies seem to define the prevalence of Dirofilariosis in the world [6]. On one hand, the prevalence and geographic range of infection with *D. immitis* are increasing in Central and Eastern European countries and in the USA [7–11]. Climate change and the spread of competent mosquito species, easy movement of dogs from one place to another, introduction of invasive, competent mosquito species, the presence of stray dogs with high prevalence of HWI, misdiagnosis and inadequate preventive treatments can explain the spread and/or increase in the prevalence of HWI in the aforementioned regions [6, 12]. On the other hand, due to increased awareness of the owners and veterinarians and widespread compliance with preventive treatments, the prevalence of HWI has decreased in endemic areas of some Western European countries and Japan [6, 12–14]. It was proposed that the reduction in the vector populations through industrialization, urbanization and insecticide treatments contributed to the decrease in the prevalence of HWI, too [6].

In Australia, canine HWI has been endemic in the states of New South Wales (NSW), Northern Territory and Queensland (QLD) with highest prevalence (up to 90%) recorded in QLD [15]. Macrocyclic lactones (MLs) were introduced for the prevention of HWI in Australia in the early 1990s (Heartgard, 1990, Dr. Phillip McDonagh, Boehringer Ingelheim Animal Health Australia, personal communication). Over the next 2 decades, reported prevalence of infection in NSW decreased from up to 50% in the 1980s to statistically zero [16–18]. In QLD, recent reports have shown a high prevalence of HWI in shelter dogs [5.8% (7/121) in Southern QLD, 8.7% (2/23) in Central QLD and 31.8% (7/22) in Northern QLD] [19], pig hunting dogs [21% (12/57) in Central QLD] [17] and wild dogs [36% (10/28) in Northern QLD] [20].

*Dirofilaria immitis* can infect humans, causing granulomas (coin lesions) in the peripheral branches of the pulmonary artery. Domestic dogs are the likely reservoir of human infection, with most infections occurring in areas where canine dirofilariosis is endemic [21, 22].

Management of HWI is complex and its success relies on accurate diagnosis [8, 23, 24]. Failure to detect infected dogs and to eliminate the worms can result in severe disease and death of the host [8, 24]. Detection of microfilariae (Mf) and circulating (unbound) antigen have been commonly used to confirm clinical diagnosis and evaluate the success of adulticide therapy and for epidemiological studies [25, 26]. Compared to detection of Mf by microscopy, antigen detection is less laborious, has higher sensitivity and specificity, and detects most ‘occult’ infections (i.e. adult worms present but no circulating microfilariae)—which have been reported in up to 67% of the infected dogs [23, 26–34]. Thus, in the last 3 decades, antigen detection has been the mainstay for heartworm testing in living dogs [23, 26–32, 35].

The sensitivity and specificity of antigen detection tests have been extensively investigated [28, 29, 35–39]. When used according to manufacturers’ instructions (no heat or chemical treatment of samples to dissociate immune complexes) the reported sensitivity of the commercially available antigen detection tests varies from 90.9 to 99.5% and specificity varies from 94 to 98.8% [28, 37]. However, sensitivity can be affected by factors including chemical treatments (mostly MLs) of the host, number of worms and composition of the worm population, and trapping of antigen (blocked antigen) in immune complexes (ICs). The latter is presumably due to presence of specific antibodies in high levels and/or having high affinity [30, 35, 40–43]. Lowered sensitivity due to blocked antigen has been recorded from numerous regions of the world and for all commercial antigen detection tests examined [28, 33, 41, 42].

Research on dogs with known composition of heartworm populations showed that dissociation of ICs by heat treatment leads to an increase in sensitivity by 7.7% and a decrease in specificity by 1.7% [41]. However, [28] showed that heat treatment of samples may improve the specificity of antigen testing by turning conventional antigen testing (CAT) false-positive results into true-negative results. Heat treatment of samples, however, increases the cross-reactivity of antibodies in *D. immitis* antigen detection tests with antigens belonging to *D. repens* and *Angiostrongylus vasorum* [44, 45] and thus reduces the specificity of the tests. This is a major concern in areas where *D. repens* and *A. vasorum* occur. Worldwide use of dissociation of ICs before antigen testing has confirmed that it improves detection of *D. immitis* antigen [23, 28, 40–42, 44, 46] and allows detection of antigen up to 1 month earlier than previously possible [47]. The clinical value of dissociation of ICs in the diagnosis of HWI is questioned by Savadelis et al. (2018) because heat treatment of samples may detect residual antigen leftover after the death of adult worms caused by long-term ML treatments, as in the ‘slow kill’ protocols [43, 48]. However, both host welfare and epidemiological studies will benefit from dissociation of ICs and improved diagnosis [42].

The dissociation of ICs was initially part of the heartworm diagnostic methodologies [24, 39, 49], but it is not routinely used in the practice environment and is not recommended by the manufacturers of the antigen detection tests, the American Heartworm Society (AHS) or the European Scientific Counsel on Companion Animal Parasites (ESCCAP) [32]. It requires a larger volume of sample and extra equipment and is comparatively both laborious and time consuming. Instead, further research was proposed to identify factors associated with ICs formation to allow general recommendations to be made on which patients would benefit most from dissociation of ICs before antigen testing [33]. Apart from the presence of microfilariae, ML treatment(s) is the only factor so far identified as being associated with blocked antigen [23, 33, 43, 50].

Because antigen and Mf detection [modified Knott’s test (MKT)] methods complement each other [23, 51–54], and because of the negative effect of the ICs on antigen detection [46], since 2014 it has been recommended to use antigen testing in tandem with Mf detection for the diagnosis of HWI and annual screening of dogs [32, 33, 55]. The use of both antigen and Mf detection is particularly important in areas with low prevalence of infection and low worm burdens. In these situations, the sensitivity of the diagnostic tests tends to decrease [1, 51]. Despite these recommendations many clinics rely only on antigen detection for diagnosis of HWI.

The objective of this study was to determine the prevalence of HWI in two geographical locations (Brisbane, subtropical area and Townsville, tropical area) of the state of QLD, Australia. Multiple diagnostic methodologies were used to optimize the detection of HWI. Possible associations among microfilaraemia, location, sex and age on formation of ICs (blocked antigen) were also explored.

## Methods

### Study population and blood sampling

The study used 383 dogs that were accepted to two animal shelters, Brisbane and Townsville, located in two widely separated geographical areas of the state of Queensland, Australia (Animal Ethics Approval numbers A2671 & ANRFA/SVS/411/16). The Brisbane shelter in the suburb of Wacol has a subtropical climate (latitude − 27.47° and longitude 153.02°). Townsville has a tropical climate (latitude − 19.15° and longitude 146.81°). Dogs transferred from other regions to the Brisbane or Townsville shelters were excluded from the study. On admission to the shelter, dogs are routinely examined physically and tested for infection with *D. immitis* by CAT. Dogs included in this study were older than 6 months, clinically healthy and with unknown heartworm prevention history.

Brisbane shelter dogs (*n* = 174) were sampled between November 2016 and March 2018 and Townsville shelter dogs (*n* = 209) were sampled between January and June 2020. The age of the dogs in both locations was determined by reading the microchip, when present, or it was estimated by examination of the dentition with categories of juvenile, young adult, adult and senior. If an age range was recorded, this was replaced by the midpoint of the range; three Townsville dogs were recorded as “2–3 years” and two Townsville dogs were recorded as “4–5 years”. Age was recorded for 173 Brisbane dogs (99.4%), with one dog without a recorded age. For Townsville, either a categorical or numeric age was recorded for 208 dogs (99.5%), with age imputed based on the category recorded for 27 dogs (12.9%) as follows: two dogs recorded as “senior” were coded as 10 years old, 14 dogs as “adult” which were coded as 5 years old, eight as “young adult” which were coded as 2 years old and three dogs recorded as “juvenile” which were coded as 6 months. Two male dogs in Townsville with discordant antigen test results (positive for antigen after dissociation of ICs) had a categorical age of “adult” imputed, nine dogs had imputed age for concordant positive results, and 16 dogs had concordant negative results.

Blood was collected in EDTA and Z Serum Clot Activator tubes (Greiner Bio-One, Kremsmünster, Austria) by the veterinary staff of the shelter. All samples were tested for HW antigen(s) both before and after dissociation of ICs and microfilariae (MKT) within 7 days of blood collection (blood samples were preserved at 4 °C from the time of collection until processing). Re-examination of samples from dogs with discordant antigen test results with a second antigen detection test was performed using samples preserved at − 20 °C for more than 3 months.

### Antigen detection

All dogs in this study were tested for antigen using unheated samples (conventional antigen testing, CAT) and heated samples (antigen testing after heat treatment, ATHT) using a commercially available antigen detection test (Witness Dirofilaria, Zoetis, Parsippany, NJ, USA). Testing without heat processing of samples was performed following the manufacturer’s instructions. Dissociation of the immune complexes by heat treatment was carried out as previously described [46]. Briefly, sera (400 ml) were heated at 104 °C in a heat block (Thermo Fisher Scientific, Waltham, MA, USA) for 10 min and allowed to reach room temperature and then the precipitate was spun down at 4000 g (ISG micro, ISG, Bexwell, UK) for 12 min (if the supernatant was not expressed the sample was centrifuged again). Forty microlitres of supernatant was loaded into the sample well of the antigen detection test (Witness Dirofilaria, Zoetis, Parsippany, NJ, USA) and the reaction was read 10 min later as per standard procedure.

Seven randomly selected sera samples (32%) from the 22 dogs with antigen test-discordant results were retested without heating to confirm the negativity of the initial results. Twenty-one sera samples (95%) from the 22 dogs with antigen test-discordant results were also analysed without heat treatment using a second antigen detection test, Anigen rapid CHW Ag Test Kit 2.0 (Bionote, Hwaseong-si, Gyeonggi-do, Korea), following the manufacturer’s instructions.

### Microfilariae detection

Samples from all dogs in this study were checked for Mf using modified Knott’s test (MKT) [56]. Briefly, 1 ml of blood collected on EDTA was mixed with 9 ml of 2% formalin and centrifuged at 500 g (Eppendorf 5702 R, Eppendorf, Hamburg, Germany) for 5 min. After the supernatant was discarded one drop of 0.1% methylene blue was added over the sediment. The pellet was examined for Mf using a bright-field microscope (Zeiss, model Axioskop 40, Jena, Germany) equipped with a digital camera (Zeiss, model AxioCam MRc, Jena, Germany). The Mfs were identified using morphometry and morphological features [57–59].

### Statistical analysis

A sample size of 149 animals was required [60, 61] assuming a 2% prevalence and a 95% confidence in both locations. The sample size was increased as the prevalence was an approximation due to lack of published studies on prevalence of HWI in Brisbane and Townsville in the last 30 years.

Variables were summarised according to their distribution and type, with categorical and binary variables as a proportion (%), normally distributed variables as mean (with standard deviation) and non-normally distributed variables as median (inter-quartile range). Test prevalence was calculated using exact Clopper-Pearson binomial intervals and prevalence compared between the two locations with two sample test of proportions with multiple-comparison Bonferroni adjustment. The McNemar’s test was used to compare the proportions of positive results for these three tests, with mid-*P* values [62] reported before and after multiple-comparison Bonferroni adjustment.

To model the association of discordant antigen test results (with a change from negative on CAT to positive after heat treatment versus no change after heat treatment) between MKT result (negative, positive), location (Brisbane, Townsville), sex (male, female) and age, a logistic regression model was fitted with the age centred at the mean of 2.5 years. The outcome variable was a discordant antigen test result, with all discordant results in this sample being negative if the sample was processed without heat treatment and a positive test if the sample was heat treated. Explanatory variables and interaction terms were added sequentially to the null model in a forward stepwise approach, with model fit assessed using likelihood ratio tests (LRT). The linear effect of age was not statistically significant; however, as the quadratic effect both terms were included. Two-way interaction terms were not significant; a three-way interaction term was found to be statistically significant for MKT result, location, sex and age, but the model appeared overfitted and is not reported. A simpler model was chosen to avoid overfitting to the sample dataset and allow clear interpretation.

Analyses were conducted in Stata version 16.1 [63] and the significance level was set at 0.05.

## Results

### Test prevalence of infection with *D. immitis* in Brisbane and Townsville locations

**Fig. 1:**
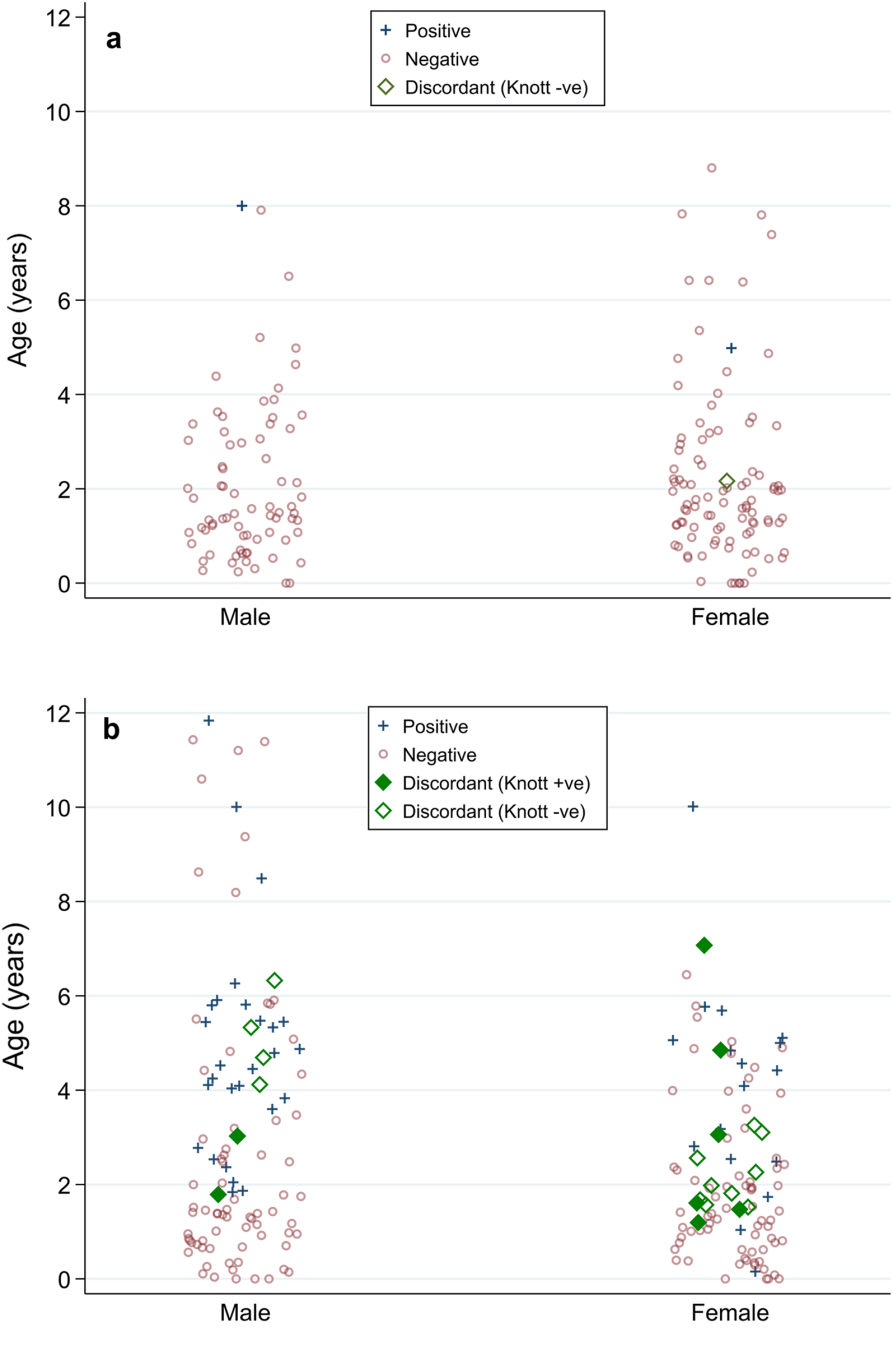
Antigen test results before and after dissociation of immune complexes by heat treatment of samples for Brisbane (**a**) and Townsville (**b**) dogs. For each shelter location positive dogs are represented by crosses and negative dogs by circles. Discordant antigen test pairs are shown as diagonals: filled diagonals represent dogs that are microfilaraemic and hollow diagonals dogs that are amicrofilaraemic

The characteristics of dogs from Townsville and Brisbane locations are given in Table 1. The median age of dogs in the trial was 2.0 years (IQR: 1.0–3.1), and there were 180 males (47.0%) and 203 females (53.0%). The test prevalence of infection with *D. immitis* was significantly higher in dogs from Townsville than in dogs from Brisbane, with Bonferroni corrected *P* value $< 0.001$ for all diagnostic tests used (CAT: Z = 6.2, MKT: Z = 5.14, ATHT: Z = 7.65) (Table 2, Fig. 1). In Brisbane 1.1% (95% CI 0.1, 4.1) of the dogs were positive for *D. immitis* by CAT, MKT and CAT and MKT combined, and 1.7% (95% CI 0.1, 5.0) were positive for *D. immitis* antigen after dissociation of ICs. In Townsville, 22% (95% CI 16.6, 28.2) of the dogs were positive for *D. immitis* by CAT, 16.7% (95% CI 12.0, 22.5) were positive by MKT, 25.8% (95% CI 20.0, 32.3) were positive by CAT and MKT combined, and 32.1% (95% CI 25.8, 38.8) were positive for antigen after dissociation of ICs (Table 2).

**Table 1:** Characteristics for dogs in the trial by shelter location

	Brisbane dogs (*n* = 174)	Townsville dogs (*n* = 209)	All dogs (*n* = 383)
Age (years), median (IQR)	1.5 (0.99–2.9)	2.0 (1.0–4.3)	2.0 (1.0–3.1)
Sex			
Male	75 (43.1%)	105 (50.2%)	180 (47.0%)
Female	99 (56.9%)	104 (49.8%)	203 (53.0%)
Missing	0 (0%)	0 (0%)	0 (0%)

**Table 2:** Test prevalence of infection with *D. immitis* by diagnostic test and shelter location

Diagnostic test	Brisbane dogs (*n* = 174)			Townsville dogs (*n* = 209)			All dogs (*n* = 383)			Prevalence difference (95% CI)^a^	*P* value ^b^
	Positive	Prevalence (95% CI)	Age (years), median (IQR)	Positive	Prevalence (95% CI)	Age (yrs), median (IQR)	Positive	Prevalence (95% CI)	Age (yrs), median (IQR)		
Conventional antigen testing	2	1.1% (0.1, 4.1)	6.5 (5.0, 8.0)	46	22.0% (16.6, 28.2)	5.0 (2.0, 5.0)	48	12.5% (9.4, 16.3)	5.0 (2.0, 5.0)	20.9% (15.0, 26.7)	< 0.001
Modified Knott’s test	2	1.1% (0.1, 4.1)	6.5 (5.0, 8.0)	35	16.7% (12.0, 22.5)	4.5 (2.0, 5.0)	37	9.7% (6.9, 13.1)	4.7 (2.3, 5.0)	15.6% (10.3, 20.9)	< 0.001
Conventional antigen testing and modified Knott’s test combined	2	1.1% (0.1, 4.1)	6.5 (5.0, 8.0)	54	25.8% (20.0, 32.3)	3.0 (2.0–5.0)	56	14.6% (11.2, 18.6)	4.5 (2.0, 5.0)	24.7% (18.5, 30.8)	< 0.001
Antigen testing after heat treatment	3	1.7% (0.1, 5.0)	5.0 (2.2, 8.0)	67	32.1% (25.8, 38.8)	4 (2.0, 5.0)	70	18.3% (14.5, 22.5)	4.0 (2.0, 5.0)	30.3% (23.7, 36.9)	< 0.001

^a^Estimated difference in prevalence between Brisbane and Townsville

^b^*P* value for two-sample test of proportions, with Bonferroni correction

### Comparison of diagnostic tests in detection of infection with *D. immitis*

Of the 383 dogs tested in the Brisbane and Townsville shelters, 48 (12.5%, 95% CI 9.4, 16.3) tested positive for *D. immitis* on CAT and 37 (9.7%, 95% CI 6.9, 13.1) tested positive for Mf on MKT (Table 2). The differences between the number of dogs that tested positive on CAT and those that tested positive on MKT were not significant after Bonferroni correction (${\chi }^{2}=\hspace{0.17em}4.48,\mathrm{ df}\hspace{0.17em}=\hspace{0.17em}1, uncorrected mid-P = 0.03, corrected P = 0.10$) (Table 3). Fifty-six (14.6%, 95% CI 11.2, 18.6) dogs tested positive for *D. immitis* on CAT and MKT combined (Table 2).

**Table 3:** Comparison of diagnostic tests in detection of infection with *D. immitis* for Townsville and Brisbane dogs combined

Diagnostic tests compared	No. of dogs with each combination of results				Estimated difference (%) (95% CI)^a^	*P* value^b^	Bonferroni corrected *P* value^c^
	+ / +	+ / −	− / +	− / −			
Antigen testing after heat treatment/conventional antigen testing	48	22	0	313	5.7% (3.2, 8.3)	< 0.001	< 0.001
Antigen testing after heat treatment/modified Knott’s test	37	33	0	313	8.6% (5.5, 11.7)	< 0.001	< 0.001
Conventional antigen testing/modified Knott’s test	29	19	8	327	2.9% (− 0.03, 5.8)	< 0.03	< 0.10
Antigen testing after heat treatment/conventional antigen testing and modified Knott’s test combined	56	14	0	313	3.7% (1.5, 5.8)	< 0.001	< 0.001

^a^Using McNemar’s test

^b^Mid-*P* value for McNemar’s test, without Bonferroni correction

^c^Mid-*P* value for McNemar’s test, with Bonferroni correction

After heat processing of samples, 22 (6.6%) out of 335 samples that had tested negative on CAT tested positive for antigen, resulting in a total of 70 (18.3%) dogs testing positive for infection with *D. immitis* (Table 2). The differences between the number of dogs positive for antigen before and after heat treatment (48 vs. 70) were significant $({\chi }^{2}=\hspace{0.17em}22.0,\mathrm{ df}\hspace{0.17em}=\hspace{0.17em}1, uncorrected mid-P<0.001, corrected P< 0.001) .$ (Table 3) The number of dogs that tested positive for antigen after heat treatment was significantly higher than the number of dogs positive for Mf by MKT as well: 70 vs. 37 dogs (${\chi }^{2}=\hspace{0.17em}33.0,\mathrm{ df}\hspace{0.17em}=\hspace{0.17em}1, uncorrected mid-P<0.001, corrected P< 0.001) .$ (Table 3).

Heat processing of samples before antigen testing detected all dogs positive by CAT and/or MKT and 14 (3.8%) dogs that reacted negative on both CAT and MKT. The number of dogs that tested positive for antigen after heat processing of samples was significantly higher than the number of dogs positive on CAT and MKT combined: 70 vs. 56 $\left({\chi }^{2}=\hspace{0.17em}14.0,\mathrm{ df}\hspace{0.17em}=\hspace{0.17em}1, uncorrected mid-P<0.001, corrected P< 0.001\right)$ (Table 3). No samples that tested antigen positive on CAT returned negative results after subsequent heat processing.

### Discordant test results

#### Conventional antigen test and modified Knott’s test-discordant results

Twenty-seven (7.0%) dogs (14 males and 13 females) had discordant results on CAT and MKT (Table 3). There were 19 (5%) dogs that tested positive on CAT but negative on MKT (12 males and 7 females), and 8 (2.1%) dogs that tested positive on MKT and negative on CAT (2 males and 6 females) (Table 3). The median age of the dogs discordant on CAT and MKT was 3 years (IQR: 2.0, 5.0). The median age of the dogs that tested positive on CAT was 5.0 years (IQR: 2.0, 5.0) while the median age of the dogs that tested positive on MKT was 4.7 years (IQR: 2.3, 5.0) (Table 2). More males than females tested positive for antigen by CAT (29 males vs. 19 females), but similar numbers of males and females tested positive for Mf (19 males vs. 18 females).

#### Antigen test-discordant results

Twenty-two (5.7%, 95% CI 3.2, 8.3) dogs (6 males and 16 females) out of the 383 dogs in the trial had antigen test-discordant results (Tables 3, 4, Fig. 1). The median age of the dogs that tested positive on CAT was 5.0 (IQR: 2.0, 5.0) years while the median age of dogs that tested positive for antigen after heat processing of the samples was 4.0 (IQR: 2.0, 5.0) years. The median age of the antigen-discordant dogs was 2.3 (IQR: 2.0 to 4.0) years compared to 1.5 (0.8–2.6) years for dogs with negative-concordant results and 5.0 (IQR: 2.0–5.0) years for positive-concordant dogs (Table 4). Eight dogs (36.4%) out of the 22 discordant dogs on antigen testing were positive for Mf while 14 (63.6%) were negative on MKT (Table 4).

**Table 4:** Characteristics of dogs with discordant (blocked antigen: positive for antigen only after dissociation of immune complexes) and concordant (positive or negative for antigen both before and after dissociation of immune complexes) antigen test results

	Discordant dogs (*n* = 22)		Concordant dogs (*n* = 361)		All dogs (*n* = 383)
	Modified Knott’s test positive (*n* = 8)	Modified Knott’s test negative (*n* = 14)	Conventional antigen testing positive (*n* = 48)	Conventional antigen testing negative (*n* = 313)	
Age (years), median (IQR**)**	2.3 (1.3–4.0)	2.3 (2.0 –4.0)	5.0 (2.0–5.0)	1.5 (0.8–2.6)	2.0 (1.0–3.0)
Sex					
Male	2 (9.1%)	4 (18.2%)	29 (8.0%)	140 (38.8%)	175 (45.7%)
Female	6 (27.3%)	10 (45.5%)	19 (5.3%)	158 (43.8%)	193 (50.4%)
Missing	0 (0%)	0 (0%)	0 (0%)	15 (4.2%)	15 (3.9%)
Location					
Townsville	8 (36.4%)	13 (59.1%)	46 (12.7%)	142 (39.3%)	209 (54.6%)
Brisbane	0 (0%)	1 (4.5%)	2 (0.6%)	171 (47.4%)	174 (45.4%)

### Logistic regression model for antigen test-discordant results

The outcome variable for the logistic regression model was a discordant antigen test result, with all discordant results in this study being a change in result from negative on CAT to positive when the sample is heat treated.

#### Location

**Fig. 2:**
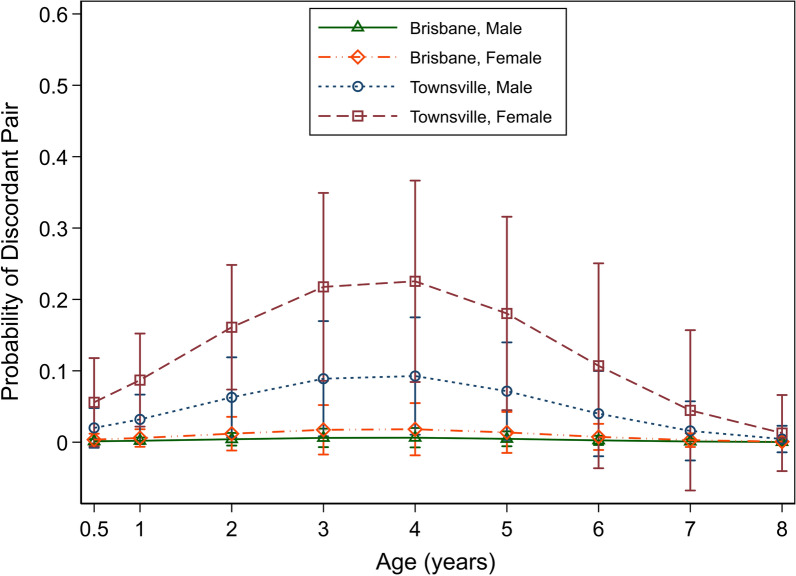
Predicted probability of a discordant antigen test result versus age, sex and shelter location

The odds of a discordant antigen result were estimated to be 16.4 times higher (95% CI 2.1–127.8) in Townsville than in Brisbane (Table 5). The estimated predicted probability of a discordant antigen test result in Townsville was 9.3% (95% CI 5.5–13.1) compared to 0.7% (95% CI 0.0–2.0) in Brisbane, after adjusting for age, sex and MKT results (Fig. 2).

**Table 5:** Logistic regression model for discordant antigen test (positive for antigen only after dissociation of immune complexes) results

	Regression coefficient (odds ratio)	95% CI	*P* value ^a^
Shelter location			
Townsville	16.4	(2.1, 127.8)	< 0.001
Brisbane	–	–	–
Age^b^ (years, centred at mean age)	1.5	(1.0, 2.2)	0.65
Age squared (years^2^, centred at mean age)	0.8	(0.7, 1.0)	0.004
MKT result			
Positive	3.2	(1.9, 9.2)	0.04
Negative	–	–	–
Sex			
Female	2.9	(1.1, 8.1)	0.03
Male	–	–	–

Baseline categories are: male, modified Knott’s test (MKT) negative, Brisbane

^a^All *P* values using likelihood ratio test

^b^Age included as age squared is significant. LRT for both age terms has a *P* value of 0.013

#### Age, sex and MKT results

There was evidence of an association between odds of a discordant antigen test result and linear effect of quadratic effect of age, sex and MKT result with *P* values of 0.004, 0.03 and 0.04, respectively (Table 5). For age, the quadratic term was statistically significant on a likelihood ratio test; the predicted probability of a discordant antigen test result rises then falls with increasing age. For a dog at age 1 year, the predicted probability is 3.8% (95% CI 1.0 to 6.5), which increases to 9.5% (95% CI 4.3–14.8) and 9.9% (95% CI: 4.5–15.4) at age 3 and 4 years, respectively, and falls to 1.9% (95% CI 0.0–6.7) at age 7 years; these are reported after adjustment for the remaining variables. Given the quadratic term for the age, the marginal probabilities are reported for the sex and MKT variables for a dog of mean age of 2.5 years, after adjustment for the remaining explanatory variables.

The odds of a discordant antigen result were 2.9 times higher (95% CI 1.1–8.1) in females than in males (Table 5). For an animal of mean age of 2.5 years, the predicted probability of a discordant antigen test result for a male dog was 3.3% (95% CI 0.7–5.7) while for a female dog it was 8.1% (95% CI 4.6–11.6) (Fig. 2).

**Fig. 3:**
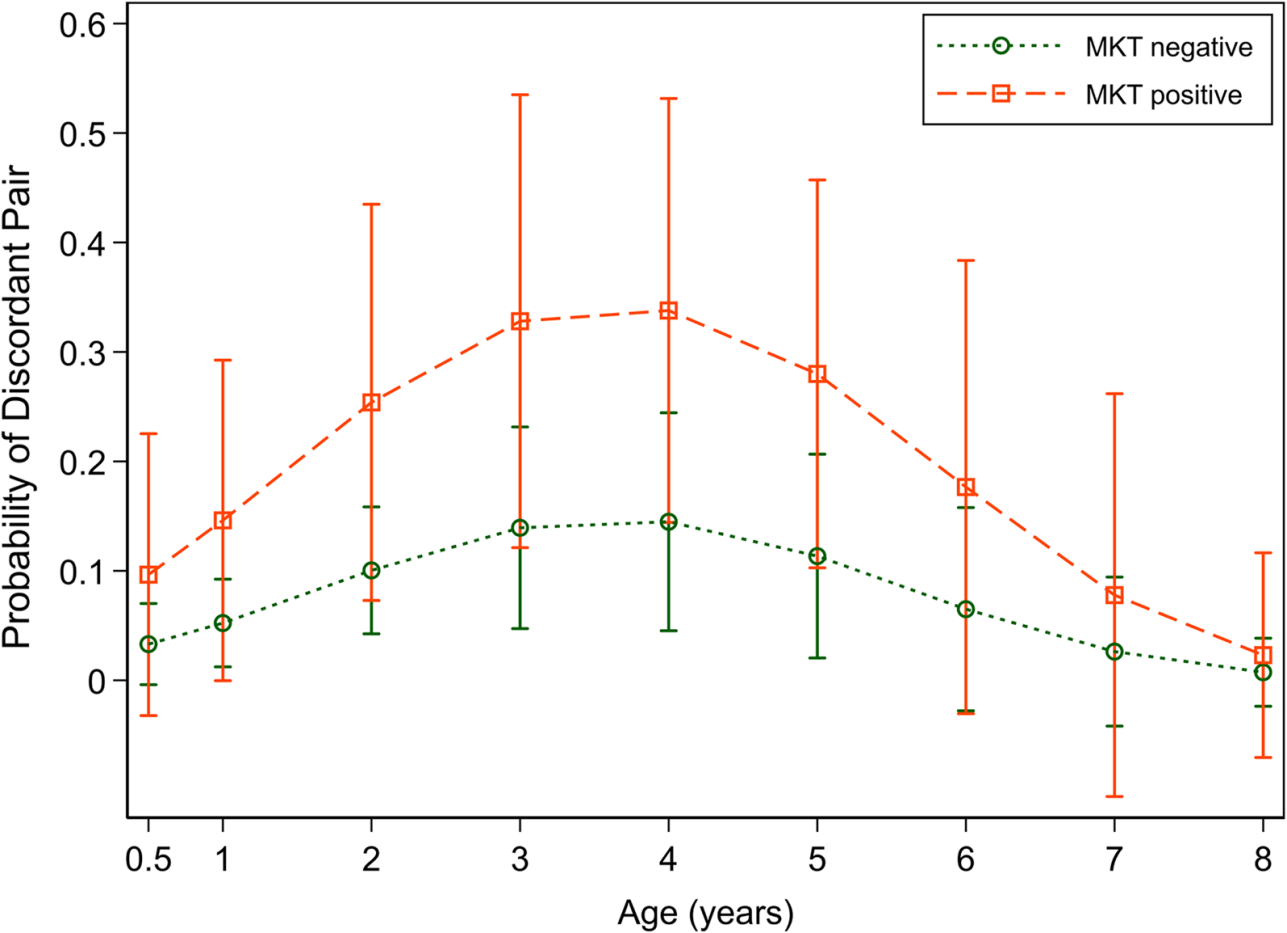
Predicted probability of a discordant antigen test result versus age by modified Knott’s test (MKT) result for Townsville dogs

The odds of a discordant antigen result were 3.2 times higher (95% CI 1.9–9.2) in MKT-positive dogs than negative dogs. For an animal of mean age of 2.5 years, the predicted probability of a discordant antigen test result of a dog who tested negative to MKT (amicrofilaraemic) was 4.5% (95% CI 2.3–6.8), with those who tested positive to MKT (microfilaraemic) having an estimated probability of a discordant antigen result of 11.7% (3.8 to 19.5) (Fig. 3).

### Reactivity of the antigen test-discordant samples with second antigen detection test

Twenty-one dogs out of the 22 dogs with discordant antigen test results were checked for HWI using a different antigen detection kit (Anigen rapid CHW Ag Test Kit 2.0). Eight dogs (38.1%), including one microfilaraemic dog, tested positive for antigen using the Anigen antigen detection test.

## Discussion

Limited studies on prevalence of HWI have been undertaken in dogs from either Townsville or Brisbane since the introduction and widespread use of MLs 30 years ago [19]. In the current study, clear differences in the prevalence of infection, and changes in prevalence compared with 30 years ago, were found between Townsville and Brisbane locations. Historically, the prevalence of infection with *D. immitis* has been higher in Townsville (tropical area) than in Brisbane (subtropical areas) [17, 64, 65].

In the Brisbane area, we found a very low prevalence of positive dogs (≤ 1.7%). This is an obvious decrease from the prevalence reported in shelter dogs in the early 1990s, 49–60% [66, 67]. A prevalence of 6.8% (6/89) was reported recently [19]. The differences in prevalence between our study and the previous one [19] may be explained by the differences in sample size in the two studies (174 dogs in our study vs. 89 in the study of [19]) or the timing of collection of the blood samples, 2016–2018 in our study and 2020 in [19] study. Alternatively, the data reported by [19] may reflect a recent increase in the prevalence of infection in the Brisbane area. This would be a major concern for veterinarians and dog owners.

The prevalence of *D. immitis* infection in dogs from Townsville (22% by CAT, 16.7% by MKT and 32.1% by ATHT) was lower than that reported in the 1970s (77%) [68] but higher than the 15% prevalence reported in 2001 [69]. The apparent re-emergence of infection with *D. immitis* in Townsville raises important questions. Many factors related to vector (geographic location, presence of competent mosquito species, relative humidity and temperature, vegetation etc.), parasite (ambient temperature) and host (owner compliance with chemoprophylaxis, ML's lack of efficacy/chemoresistance, socio-economic status of dog owners, presence of wild canid reservoirs, etc.) can affect the prevalence of infection with *D. immitis* and may have contributed to the differences in HWI prevalence found between Brisbane and Townsville.

Two major events that occurred recently in Townsville might have impacted the size and composition of populations of mosquito vectors: the release in 2015–2016 of *Wolbachia* transinfected *Aedes aegypti* mosquitoes [70] to control Dengue fever and the unprecedented floods that occurred in January–February 2019. Analysis of data on populations of *Culex annulirostris* and *Aedes vigilax* trapped weekly from 23 August 2017 up to start of this trial (January 2020) (kindly provided by Scott Dunsdon: Townsville City Council and Scott Lyons: Townsville Public Health Unit) suggests that these events did not have a substantial effect on the population sizes of these two mosquito species. However, this requires further investigation as neither of the mosquito species trapped in Townsville are very efficient vectors for *D. immitis* and the competent vectors for *D. immitis* in Queensland are not well studied [71–73].

Development of *D. immitis* within the mosquito vector is temperature dependent and it does not occur at temperatures lower than 14 °C [74]. The amount of heat required to reach the infective stage (L3) can be expressed in degree days or heartworm development units (HDUs) in excess of the 14 °C threshold temperature [74, 75]. On average, a total of 130 °C/HDUs over at most 30 successive days are required for microfilariae to reach the infective stage [75, 76]. Calculation of HDUs [74] for the year 2020 (daily temperatures downloaded from Bureau of Meteorology, Australian Government, http://www.bom.gov.au/) has shown that in Townsville *D. immitis* may develop within the vector all year around while in Brisbane the development is likely to be halted during the cool months of winter (unpublished data).

MLs are effective and safe drugs available in a wide variety of formulations and they have been available for prevention of HWI for almost 30 years [77]. Most dogs that become positive to HWI have either not been on prevention or experienced gaps in prevention due to failure of owners to give the preventive drugs according to the product label [78, 79]. Veterinarians from Townsville and Mackay veterinary practices expressed concern about owner compliance with the preventive regimes as a potential explanation for the apparent increase in prevalence of HWI in the area (personal communication). However, a recent study involving veterinary practices from all over Australia suggested that poor compliance with HW prevention occurs all over Australia [78]. Although poor owner compliance might be a factor contributing to increased prevalence of HWI in Townsville, the level of owner compliance with preventative treatments (including those for relinquished dogs that are accepted to the shelters) seems unlikely to vary substantially between Townsville and other regions of Australia [78].

Wild dogs (dingos, dingo/domestic dog hybrids and domestic dogs living in the wild) are ubiquitous across all habitats in northern Australia [80, 81]. They are common in and around Townsville and are very often infected with parasites, including *D. immitis* [69, 81]. Data on limited numbers of wild dogs investigated for HWI in Townsville show that prevalence of infection with *D. immitis* has remained high in the last 20 years: 75% (15/20 based on necropsy [69]), 40.7% (11/27, 2007–2008, CAT, Constantinoiu, Coleman and Goullet, unpublished data) and 14% (1/7, 2018, CAT, Constantinoiu, Coleman and Goullet, unpublished data). This is in agreement with the high prevalence of HWI reported in wild dogs in Northern Queensland: 36% (10/28 based on CAT and MKT or necropsy [20] and 46% (18/39 CAT and MKT or necropsy [82]. This suggests that wild dogs in/around Townsville might serve as a reservoir of infection for urban dogs [20, 69], especially if their presence is coupled with low owner compliance with preventive regimes. In contrast, the number of wild dogs around Brisbane is lower than around Townsville [81] and the prevalence of infection with *D. immitis* in the wild dogs around Brisbane has been recorded as zero (0/40 dogs investigated by necropsy) [83].

ML chemoresistance, confirmed in USA [84] and suspected in Mackay, a city near Townsville [85], could be another cause of high prevalence of HWI in Townsville. Currently, the Mf from this trial are being sequenced in a trial investigating ML chemoresistance.

We hypothesize that the high population of wild dogs in Townsville and surroundings, potentially infected with *D. immitis* in large numbers, maintains a reservoir of infection for urban dogs in Townsville. This, combined with the worm’s ability to develop within mosquitoes all year around and possibly reduced owner compliance with preventive regimes, might have contributed to the high prevalence of HWI in Townsville. Local practitioners suspect loss of efficacy (LOE) of ML chemicals and, if confirmed, this would be a further factor. Investigating LOE was outside the scope of the current study.

The number of dogs positive on CAT was higher than the number of dogs positive for Mf (MKT) and this confirms that 19 (5%) dogs in the trial had ‘occult infections’ (i.e. adult worms present but no circulating microfilariae). This is consistent with previous data [33] and supports the widespread use of CAT as a primary diagnostic method for HWI. However, eight dogs (2.1%) tested negative on CAT but positive on MKT, reinforcing the importance of using both tests in detection of HWI as recommended by AHS [32]. As all eight microfilaraemic dogs tested positive for antigen after heat treatment it is very likely that *D. immitis* antigen in these dogs was blocked in immune complexes. More males than females tested positive on CAT (29 vs. 19). However, similar numbers of males and females tested positive for antigen after heat treatment or for MF by MKT. This suggests that the formation of immune complexes may be more common in female dogs.

Dissociation of ICs by heat processing of the samples detected all dogs positive by CAT and/or MKT as well as dogs that were negative on both diagnostic tests. Heat treatment of the samples increases the cross-reactivity of antibodies in *D. immitis* antigen detection tests with antigens belonging to *D. repens* and *A. vasorum* [44, 45]. However, this is unlikely to be the case here as *D. repens* does not occur in Australia and *A. vasorum* is extremely rare, with only 1–2 cases ever reported [86].

The number of dogs that tested positive for antigen after heat treatment was significantly higher than the number of dogs positive on either CAT, MKT or CAT and MKT combined. Overall, 6.6% (22/335) of the samples [0.6% (1/172) for Brisbane and 12.9% (21/163) for Townsville] had *D. immitis* antigen blocked in immune complexes and changed from antigen-negative status on CAT to positive status after heat treatment. This is consistent with previous research that showed that between 5.2% and 20.2% of samples change from initial antigen negative to antigen positive status after heat processing [23, 33, 40, 42, 44]. Our data are at odds with data reported by [19] that found only one sample (0.7%) out of 152 plasma samples changed the status from antigen negative to antigen positive after heat treatment.

It is assumed that ELISA technology provides superior sensitivity than lateral flow immunochromatography for the diagnosis of HWI [33, 36]. The diagnostic tests used in these trials (DiroCHEK, ELISA [19], Witness Dirofilaria, lateral flow immunochromatography, present trial) are based on different technologies but both have very high diagnostic sensitivity (Se) and specificity (Sp): DiroCHEK, Zoetis (Se 100%, Sp 100%) and Witness Dirofilaria, Zoetis, (Se 97%, Sp 96%) and 98.8% positive agreement [28, 36, 37, 41, 87, 88]. Diagnostic sensitivity of both tests decreases in samples coming from low heartworm burden dogs [36].

Frequency of blocked antigen mirrors prevalence of HWI [23]. Our data support this finding; in the low prevalence area of Brisbane we found only one antigen-discordant sample (0.6%) out of the 172 CAT-negative samples tested while in the high prevalence area of Townsville we found 21 antigen-discordant samples (12.9%) out of the 163 CAT-negative samples tested. The odds of an antigen-discordant test result were 16.4 times higher in samples coming from Townsville than in Brisbane samples. Thus, the number and origin of the samples might explain the differences between our study and that of [19]: in the present study 209 samples came from the high-prevalence North Queensland region vs. 22 in the study of [19].

Samples were used undiluted in our study [33, 42, 44, 47, 50, 89] but diluted 1:1 in phosphate-buffered saline (PBS) in the study of [19]. Dilution of samples 1:1 in buffers containing chelating agents (0.1 M EDTA) has been validated and allows detection of antigen after heat dissociation of ICs, even in dogs with low amounts of antigen (< 5 worms) [40, 49]. However, dilution of plasma samples with PBS or 0.9% saline has not been investigated, especially in dogs with low worm burdens [23].

The lower diagnostic sensitivity of the test (Witness Dirofilaria) used in the present study with unheated samples coming from low worm burden dogs (< 10) than that of DiroCHECK (71% vs. 78%) [36] might also explain the differences between the two studies. Generally, low worm burdens are typical of populations of dogs infected with *D. immitis* [35, 36]. Currently, this seems to be the case with *D. immitis* in Townsville dogs as well: in the last 10 months, 6 of the 12 dogs found infected with *D. immitis* by post-mortem examination had 1–5 worms, 2 had 7–8 worms and 4 had > 17 worms (Constantinoiu and Taylor, unpublished data).

In this sample, dissociation of ICs decreased the median age of antigen-positive dogs from 5 to 4 years. The median age of antigen test-discordant dogs was lower than that of antigen test-concordant dogs (2.3 vs. 5 years) with dogs of 3–4 years of age having the highest odds of antigen test-discordant results. This suggests that blocked antigen is more common in younger dogs. The high levels of specific antibodies present in the early stages of infection [47, 90] might explain the high prevalence of blocked antigen in younger dogs. Dogs discordant on CAT and MKT also had a low median age, 3.0 years. Dogs younger than 3 years are generally infected with fewer worms and are amicrofilaraemic or have low numbers of Mf in the blood [52, 65, 66] and this might explain the young age of dogs discordant on CAT and MKT.

In this trial, the probability of having blocked antigen was higher in female dogs than in male dogs [in females the odds of a discordant antigen result were estimated to be 2.9 times higher (95% CI 1.1–8.1) than in males]. The differences in worm burden between female and male dogs were not significant in previous studies [66] and it is unknown why the probability of having blocked antigen was higher in females. However, this may be related to the age profile of the animals in this trial: female dogs were younger than the male dogs. Alternatively, these differences might be linked to the differences in the immune response mounted by female and male subjects as demonstrated in/with other pathogens and host species [91, 92].

Blocked antigen is more likely to occur in microfilaraemic dogs and this has been reported previously [23]. A higher antibody response occurs in microfilaraemic dogs compared with amicrofilaraemic ones [66, 93]. The probability of having blocked antigen was higher in dogs from Townsville than in dogs from Brisbane reflecting the higher prevalence of infection in the tropical city.

The antigen detection test used in this trial (Witness Dirofilaria) was chosen based on its high sensitivity and specificity [28, 37] and wide use in Australia, including in animal shelters [37, 85]. However, because some false antigen-negative results might be due to differences in the epitopes recognized by the capture antibodies used by a particular test, technology used, analytical sensitivity or intrinsic features of the test rather than the presence of blocked antigen, 21 (95%) out of the 22 antigen-discordant samples were checked without heat treatment with a different antigen detection test [Anigen Rapid Canine Heartworm (CHW) Ag 2.0 Test Kits] that has a similar sensitivity (99.5%) and specificity (94%) to the test (Witness Dirofilaria) used in this trial [37]. Eight (38.09%) out of the 21 antigen-discordant samples tested positive on the second antigen test (one out of the eight samples had circulating Mf). Therefore, our data suggest that some of the antigen-negative samples that became positive after heat dissociation of the ICs might test positive without heat treatment if evaluated with a different antigen detection test. This suggests that double checking of antigen-negative samples from dogs with suspected *D. immitis* infection using a different antigen test might be beneficial for accurate diagnosis of HWI.

The limitations of this study include the lack of confirmation of HWI by necropsy, the gold standard for diagnosis or echography [35], the testing in the two locations in different time periods and the possible changes in the prevalence of infection with *D. immitis* in the intervening time. The fact that for a minority of dogs the age was determined by examining the dentition might have influenced some of the results presented in this research. The small number of positive dogs in Brisbane and the unknown history of ML treatments are also acknowledged.

## Conclusions

Infection with *D. immitis* remains a common problem in Townsville dogs, and its prevalence seems to be increasing. This poses a serious risk to the health of susceptible hosts, including humans in the area. Furthermore, dogs from Townsville can spread the HW to new areas [94]. In Brisbane, HWI is less common but still poses a risk to canine health. For the diagnostic test used in this study (Witness Dirofilaria) dissociation of ICs before antigen testing increased the detection of Dirofilariosis (detected all dogs positive by either CAT or MKT and dogs that are negative to both CAT and MKT) with no reduction in specificity. In this study, dogs 3–4 years of age, female, microfilaraemic and from Townsville had the highest odds of blocked antigen.

## Data Availability

Small amounts of frozen sera are available from most of the samples used in this project.

## Abbreviations

ATHT: Antigen testing after heat treatment
CAT: Conventional antigen testing
HDU: Heartworm developing units
HWI: Heartworm infection
ICs: Immune complexes
MLs: Macrocyclic lactones
Mf: Microfilariae
MKT: Modified Knott’s test
NSW: New South Wales
QLD: Queensland
